# OR6-006 – IL36RN alleles in skin auto-inflammation

**DOI:** 10.1186/1546-0096-11-S1-A101

**Published:** 2013-11-08

**Authors:** F Capon, N Setta-Kaffetzi, D Berki, S Mahil, A Navarini, VM Patel, C Siew-Eng, AD Burden, C Griffiths, M Seyger, R Trembath, C Smith, J Barker

**Affiliations:** 1 King's College London, London, UK; 2 Hospital Sultanah Aminah, Johor Bahru, Malaysia; 3 University of Glasgow, Glasgow, UK; 4 University of Manchester, Manchester, UK; 5 Radboud University Nijmegen Medical Centre, Nijmegen, the Netherlands; 6 Queen Mary University of London, London, UK

## Introduction

Our group identified recessive mutations of the *IL36RN* gene in patients affected by Generalised Pustular Psoriasis (GPP), a severe skin disorder characterised by acute episodes of pustulation, fever and systemic upset.

## Objectives

Here, we sought to characterise the phenotypic spectrum associated with *IL36RN* alleles. We specifically investigated the possibility that *IL36RN* defects may also contribute to chronic forms of pustular psoriasis and to common plaque psoriasis (also known as psoriasis vulgaris or PV).

## Methods

We screened a total of 598 patients affected by GPP (n=84), or by localised forms of pustular psoriasis (9 cases of Acrodermatitis Continua of Hallopeau and 139 cases of Palmoplantar Pustulosis) and PV (n=366).

## Results

We found *IL36RN* recessive mutations in patients with GPP (7/84) and localised pustular psoriasis (2/9 cases of Acrodermatitis Continua of Hallopeau and 3/139 cases of plamar-plantar pustulosis), but not among PV cases. Of note, we also identified several affected individuals who carried a single heterozygous mutation. In fact, we uncovered a significant enrichment of heterozygous *IL36RN* alleles among patients with pustular psoriasis (frequency in cases 1.4% vs. 0.3% in controls, P=0.004), but not among subjects with PV (0.4% cases vs. 0.3% controls, P=0.77).

## Conclusion

We have demonstrated a significant overlap in the genetic basis of acute generalised and chronic localised forms of pustular psoriasis. The recurrence of similar mutations in both disease groups and the observation of affected individual carrying a single recessive allele suggest that other genes may modify the phenotypic expression of *IL36RN* variants.

Our findings argue against the notion that *IL36RN* alleles may contribute to PV. In the light of recent data suggesting a pathogenic role of IL-36 in a mouse model of the disease, our results emphasize the importance of genetic studies in the molecular dissection human auto-inflammatory diseases.

## Disclosure of interest

None declared

